# Imaging-Genomics in Glioblastoma: Combining Molecular and Imaging Signatures

**DOI:** 10.3389/fonc.2021.699265

**Published:** 2021-07-06

**Authors:** Dongming Liu, Jiu Chen, Xinhua Hu, Kun Yang, Yong Liu, Guanjie Hu, Honglin Ge, Wenbin Zhang, Hongyi Liu

**Affiliations:** ^1^ Department of Neurosurgery, The Affiliated Brain Hospital of Nanjing Medical University, Nanjing, China; ^2^ Institute of Neuropsychiatry, The Affiliated Brain Hospital of Nanjing Medical University, Fourth Clinical College of Nanjing Medical University, Nanjing, China; ^3^ Department of Neurosurgery, Institute of Brain Sciences, The Affilated Nanjing Brain Hosptial of Nanjing Medical University, Nanjing, China

**Keywords:** glioblastoma, radiomics, imaging genomics, artificial intelligence, machine learning, deep learning

## Abstract

Based on artificial intelligence (AI), computer-assisted medical diagnosis can scientifically and efficiently deal with a large quantity of medical imaging data. AI technologies including deep learning have shown remarkable progress across medical image recognition and genome analysis. Imaging-genomics attempts to explore the associations between potential gene expression patterns and specific imaging phenotypes. These associations provide potential cellular pathophysiology information, allowing sampling of the lesion habitat with high spatial resolution. Glioblastoma (GB) poses spatial and temporal heterogeneous characteristics, challenging to current precise diagnosis and treatments for the disease. Imaging-genomics provides a powerful tool for non-invasive global assessment of GB and its response to treatment. Imaging-genomics also has the potential to advance our understanding of underlying cancer biology, gene alterations, and corresponding biological processes. This article reviews the recent progress in the utilization of the imaging-genomics analysis in GB patients, focusing on its implications and prospects in individualized diagnosis and management.

## Introduction

Glioblastoma (GB) is the most common malignant primary brain tumor in adults, accounts for 48.3% of malignant brain tumors (1). Much work has focused on GB, given that it has the characteristics of infiltrating the surrounding brain tissue (2), and has an aggressive course and extremely grim prognosis (3). Gene expression profiling has provided new perspectives for the identification of tumor-associated genetic mutation, biomarkers, and therapeutic targets of glioblastoma (4, 5). The 2016 central nervous system (CNS) tumor classification of the World Health Organization (WHO) incorporated molecular markers into traditionally histopathological classification, providing an integrated phenotypic and genetic classification (6). Recently, based on transcriptomic profiling, GBs have been divided into proneural, classical, and mesenchymal subtypes (7).

The characteristic neuroradiological findings raise the initial suspicion for GB and determine the initial surgical approach and plan. Diagnosis is confirmed through histo-molecular evaluation of the tissue post-biopsy/resection. Traditional magnetic resonance imaging (MRI) has greatly improved the diagnostic efficiency of intracranial lesions, but there are still limitations in the definitive diagnosis of brain tumors. Over the past few decades, there has been a rapid development towards the application of quantitative parametric MRI techniques for GB evaluation (8). More recently, advanced computer technology has permeated the field of modern medicine, making precision medicine an inevitable trend in the development of clinical medicine. Artificial intelligence (AI) is a broad term that covers a wide range of disciplines including computer vision, machine learning, and more. Deep learning is a branch of machine learning that uses artificial neural networks as a framework to learn and characterize databases. There has been growing interest in the application of AI techniques to neuroimaging research, making machine learning and deep learning algorithms critical to the radiomics procedures.

Radiomics utilizes high-throughput radiomics features and mathematical models to quantify tumor characteristics, allowing the non-invasive capture of microscale information hidden within medical imaging (9, 10). As an important branch of radiomics, imaging-genomics (also known as radiogenomics) further links imaging characteristics (phenotypes) with genetic, mutational, and expression patterns (11, 12). The basic hypothesis of imaging-genomics is that the expression of a specific set of genes or molecular alterations affects the extractable imaging phenotypes (13). A particular focus of imaging-genomics analysis has been on the association between imaging characteristics and gene expression patterns, including the expression of individual genes and the expression of specific gene subgroups (14). Just like the resolution and complexity that genomics has brought to tumor biology, imaging-genomics also brings similar effects to traditional medical images. The ultimate goal of radiomics is to maximize the utilization of medical imaging information to help clinicians make clinical decisions, so as to minimize the possibility of potential invasive maneuvers. The increasing importance of genetic markers has also led to the rapid growth of GB imaging-genomics research, with progressively greater complexity, including the combination of artificial intelligence (AI) technology. More recently, deep learning using convolutional neural networks has further improved radiogenomics prediction (15, 16), providing unique opportunities and challenges for the diagnosis and treatment evaluation of GB.

Herein, we will review the latest researches on radiomics and imaging-genomics of GB. First, we briefly introduced the genomic characteristics of GB and the principle of imaging-genomics, and then summarized the findings of the latest research. Finally, we will present our opinions on its current challenges, and potential future directions.

## Genomics in Glioblastoma

Traditionally, GBs can be divided into “primary” and “secondary” GBs. The vast majority of GB (about 90%) develop rapidly *de novo* in elderly patients and are mostly isocitrate dehydrogenase (IDH) wild-type. While secondary GBs develops from a preexisting lower-grade glioma in younger patients and usually carry mutations in IDH (17). Radiologically, primary GBs are widely distributed in the brain, while secondary GBs are preferentially located in the area of the frontal lobe surrounding the rostral extension of the lateral ventricle in younger patients (17, 18). Primary and secondary GBs are largely histologically indistinguishable, but they are different in genetic and epigenetic profiles (17). The genomic and transcriptome characteristics of these tumors have revealed the key alterations that may contribute to the classification and evaluation of the disease (4, 19, 20) (17) (21).Different bioinformatics algorithms have identified several genes as significantly mutated in GB, including but not limited to epidermal growth factor receptor (EGFR), TP53, PTEN, Neurofibromatosis 1 (NF1), and RB1 (20, 21). Additionally, genomic analysis based on multiple sampling from the same neoplasm suggests that transcriptome subtypes are heterogeneous in GB (22) (23). Patient-specific intratumor heterogeneity of GB underlies variation in response to treatment, which contributes to the eventual failure of treatment (22, 24, 25), including drug resistance, radiotherapy resistance, and fast and incurable tumor recurrence. Thus, relying only on single tissue samples from individual patients to understand the cancer dynamics of GB is challenging.

The whole-genome sequence analysis has revealed the genetic and epigenetic landscapes of human brain GB (4, 20). Complex gene interaction events and molecular modulation networks are involved in the occurrence and development of GBs (4, 5, 26). It is expected that one or several biomarkers will provide information to help clinicians make the diagnosis, and enable clinical experts to make the best choice among various treatment options, such as surgery and/or chemotherapy, and/or radiotherapy, and/or targeted therapy. Numerous studies have identified alterations of several core signaling pathways in GB, including the RB1 pathway, the TP53 pathway, and the PI3K/PTEN pathway (4, 21). The heterogeneity of GB cells (26) suggests that targeting strategies based on multiple elements of different signaling pathways might constitute a more efficient therapy for GB (5).

Given its invasive and infiltrating nature, GB is essentially considered to be a disease of the entire brain (27). Surgical biopsy is currently the standard procedure for providing the genomics information of GB. However, a biopsy is not only highly invasive, but its accuracy is also limited by the biopsy site, resection type, and disease variability. Compared with traditional genomic analysis, imaging-genomics has the advantage of assessing the entire tumor volume, and non-invasively provides a “virtual biopsy” by predicting specific gene status, biological processes, and even core signaling pathways of GB (28–30) (28, 29, 31). High-resolution imaging technologies can present the tumor completely and three-dimensionally. Based on this property, imaging-genomics can associate the radiological phenotypes (such as shape or texture) of a specific spatial location (such as necrosis, high perfusion, or even more subtle subregions) with the tumoral molecular characteristics of the same location. Also, by associating the genetic status of the biopsy location with the quantitative imaging features, imaging-genomics has the potential to help characterize tumoral genetic heterogeneity (30), which shows diagnostic value under the context of individualized oncology.

## Radiomics and Imaging-Genomics of Glioblastoma

Traditional pathology and radiology have been primarily focused on the associations between histopathologic and visual imaging findings, while radiomics and imaging-genomics focus on exploring the relationships between imaging phenotypes and biological characteristics (11, 12). Generally, radiomics is dedicated to discovering the biological significance (such as predicting survival and treatment prognosis) of specific imaging phenotype, which when correlated with genomics is termed imaging-genomics. Imaging-genomics analysis helps to understand the biological associations behind image phenotypes, explain how biological processes are reflected in imaging, and define imaging markers associated with molecular biological characteristics. When constructing an imaging genomic map of GB, several dimensions need to be considered, including the disease course, imaging category, lesion delineation, features extraction and selection, and biological data type, as well as model building or integration (11). Typically, the imaging features used in a radiomics or imaging-genomics study are mainly divided into two categories, namely manually defined features and deep learning features.

### Feature Extraction, Selection, and Corresponding Modeling

#### Manually Defined Features

**Figure 1:**
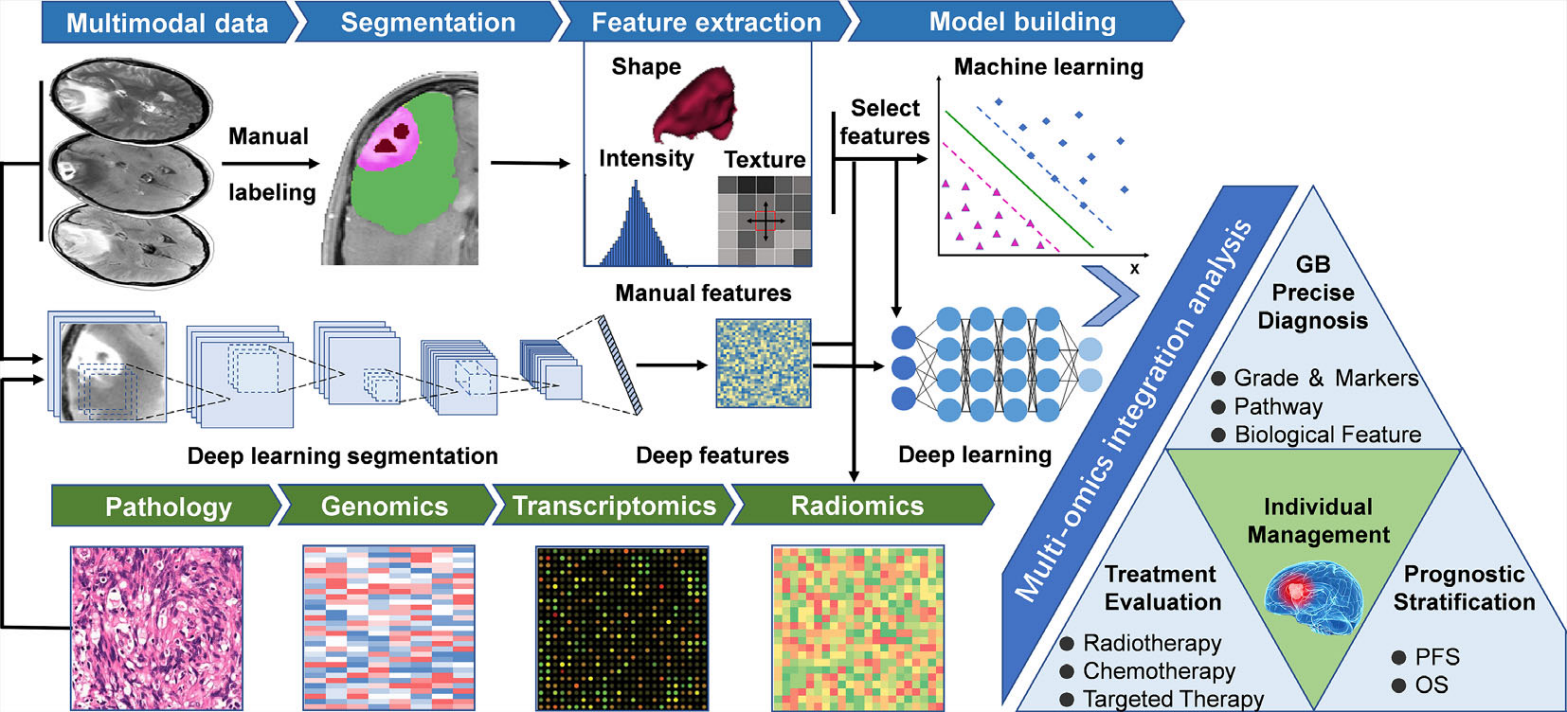
General workflow of radiomics studies in neuro-oncology. The workflow of a radiomics study, including the following steps: (1) Multimodal imaging and biological data acquisition; (2) Data preprocessing and standardization; (3) Delineation of regions of interest, including manual segmentation and deep learning-based segmentation; (4) Radiomics feature extraction using predefined algorithms or deep learning techniques; (5) Data analysis, feature reduction, and/or selection for further analysis of machine learning and/or deep neural networks; (6) Multi-omics and clinical information integrated model training and testing, guiding individualized disease diagnosis, treatment evaluation, and prognosis prediction. GB, Glioblastoma; OS, Overall Survival; PFS, Progression free survival.

Manually defined feature-based imaging-genomics utilizes specific mathematical algorithms to extract features from pre-depicted regions of interest (ROI) (**Figure 1**). After feature extraction and feature selection, machine learning models are used to solve classification and/or regression problems. Usually, the steps of the process mentioned above includes a few phases as following: 1) Data acquisition; 2) Image pre-processing, including multiparametric imaging registration, noise reduction, intensity and/or orientation normalization, spatial resampling, and corrections of MRI field inhomogeneities; 3) Segmentation of ROI, segment the tumors into necrosis, enhancement, edema, and peritumoral parts, etc. by manual delineation or (semi-) automated algorithms; 4) Feature extraction, extract different kinds of quantitative features that reflect the heterogeneity of tumors, including shape features, histogram-based features, textural features, and higher-order statistics features; 5) Feature selection, including supervised and unsupervised methods to overcome the issue of redundancy and overfitting that may be seen with multitudinous features; 6) Model building and evaluation, build a mathematical model (such as regression models, random forest (RF), and/or support vector machines (SVM) based on the selected features to predict a known, underlying ground truth, including specific genetic mutation or pathway alterations. In general, training set data is used for learning to fit the parameters of a classifier, while the validation set is used to tune the parameters of the model. Ideally, after model training and validation, the final models should be applied to the test set (unexposed independent data) to evaluate the performance of the final model. A robust radiomic model must be validated by independent, external data to show its value for clinical promotion and application.

#### Deep Learning-Based Features

Deep learning, a subcategory of machine learning based on neural networks (32), represents a class of algorithms that use stacked neural network structures (33). In deep learning-based imaging-genomics, different network structures such as auto-encoders or convolutional neural networks (CNNs) are utilized to mine the hidden features from the initial data (15, 34). The typical way to extract deep learning features is to train a deep learning model using observed indicators, and then extract the outputs of specific layers as deep learning features (35). For example, the results of the final fully connected layer of CNNs or the final convolutional layer of auto-encoder can be extracted as deep learning features (36). The extracted deep features can either be trained by an artificial neural network for classification and prediction (15) or leave the network and fit into different classifiers (35) (such as SVM, RF) to generate a model (**Figure 1**), the latter process is similar to the approach of manually defined feature-based imaging-genomics. Recently, deep learning has been successfully applied in digital pathology to segment nuclei and for the classification of molecular markers (37, 38). Deep learning-based imaging-genomics usually requires relatively large datasets. Auto-encoders provide an alternative method to extract deep features when there is not enough data to train a deep learning model (34, 36). Another technique to overcome this limitation is transfer learning (39, 40), which essentially uses a pre-trained network (usually on natural images) to solve the data set needs of deep network training (34). **Figure 1** describes the main workflow of the above two methods, and **Table 1** summarizes several radiomics studies designed according to this workflow.

**Table 1:** Applications of radiomics for predicting specific molecular markers in GB.

Study	Imaging Modality	Molecular signature(s)	No. of Patients Training + Testing	No. of features Initial + Final		Performance AUC/Accuracy	Features selection and Model building
**Li et al. (** 41 **)**	MRI, T1+T1CE+T2+Flair	IDH1 status	225 (118 + 107)	1614	8	0.96^a^	RF-Boruta, RF
**Chang et al. (** 42 **)**	MRI, T1+T1CE+T2+Flair	IDH1,MGMT,and 1p/19q	259 (80%+20%)	NA	NA	(IDH1) 94%^b^	CNNs
**Zhang et al. (** 43 **)**	MRI,T1+T1CE+T2+ Flair+DWI	IDH1/2	120 (90 + 30)	2970	387	0.92^a^	Correlation coefficient, RF
**Li et al. (** 44 **)**	PET	IDH1/2	127 (84 + 43)	1561	11	0.90^a^	Lasso, Multivariate logistic regression
**Wei et al. (** 45 **)**	MRI, T1CE+Flair+DWI	MGMT	105 (74 + 31)	3051	13	0.90^a^	Correlation coefficient, MRMR, Logistic regression
**Li et al. (** 46 **)**	MRI, T1+T1CE+T2+Flair	MGMT	193 (133 + 60)	1705	6	0.88^a^	Correlation coefficient, RF-Boruta, RF
**Qian et al. (** 47 **)**	PET	MGMT	86 (59 + 27)	1450	3	80%^b^	Correlation coefficient, RF Extra trees, SVM, Neural network
**Su et al. (** 48 **)**	MRI, T1+T1CE+T2+Flair	H3 K27M	100 (75 + 25)	85	10	0.85^a^	TPOT
**Choi et al. (** 15 **)**	MRI, T1CE+T2+Flair	IDH1/2 status	1166 (727 + 439)	NA	NA	0.96^a^	CNNs, RF
**Jin et al. (** 38 **)**	H&E Pathological slides	IDH1/2 and 1p/19q	323 (267 + 56)	NA	NA	87.6%^b^	CNNs
**Liu et al. (** 49 **)**	H&E Pathological slides	IDH1/2 status	200 (6:1:1)	NA	NA	0.931^a^	CNNs

CNNs, Convolutional neural networks; DWI, Diffusion Weighted Imaging; Flair, Fluid-attenuated inversion recovery; H&E, haematoxylin and eosin; IDH, Isocitrate dehydrogenase; Lasso, Least absolute shrinkage and selection operator; MGMT, O (6)-methylguanine-DNA methyltransferase; MRI, Magnetic resonance imaging; MRMR, Minimum redundancy and maximum relevance algorithm; NA, Not available; PET, Positron emission tomography; 1p19q, the co-deletion status of the 1p/19q chromosome arms; RF, Random forest; SVM, Support vector machines; T1, T1-weighted MRI; T2, T2-weighted MRI; T1CE, contrast-enhanced T1-weighted MRI; TPOT, the Tree-based Pipeline Optimization Tool.

The values in the performance column were achieved using the best model in the test set. ^a^ and ^b^ are used to mark the AUC and accuracy values, respectively.

### Imaging-Genomics of Glioblastoma

As a subclass of radiomics, imaging-genomics focuses more on the relationships between imaging characteristics of a disease, and its gene expression patterns, gene mutations, and other genomic-related characteristics (11). The gene expression patterns for constructing these relationships include not only the expression of a single gene (45, 50), but also the measures that summarize expressions of specific gene subsets (14, 29, 51, 52), the corresponding signal pathways (29, 53), and consequent biological effects (28, 54). ‘Imaging-genomics’ research also refers to a study aimed at discovering those relationships.

**Figure 2:**
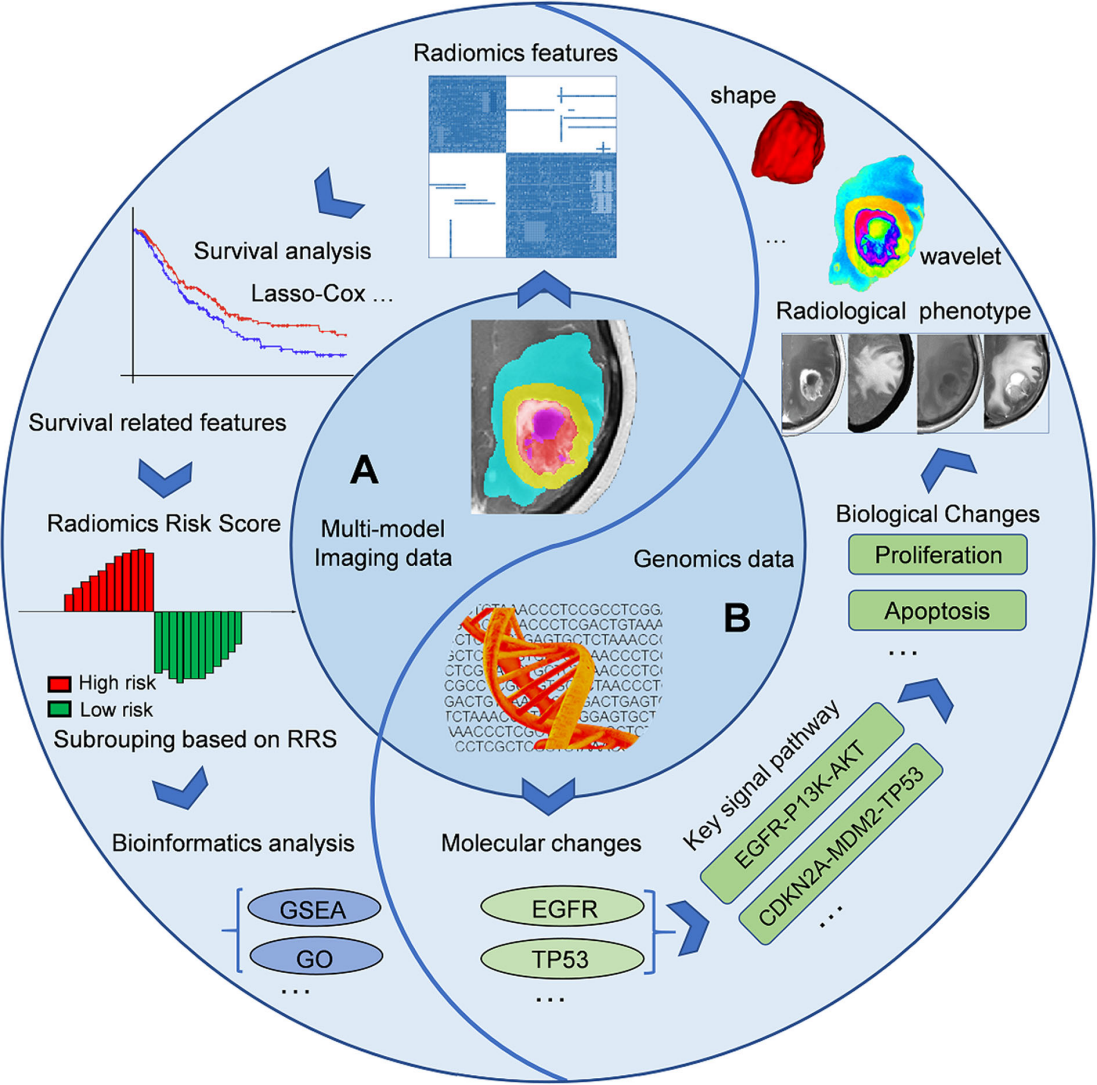
General workflow of imaging-genomics studies. A imaging-genomics research can generally build the relationship between imaging phenotypes and genetic characteristics from two perspectives. **(A)**, Left semicircle, from imaging phenotypes to genomic characteristics: using features extracted from the sub-areas of tumors in multi-modal images to construct individualized RRS or divide patients into different subgroups, and utilize the corresponding sequencing data for further bioinformatics analysis (such as GO or GSEA). **(B)**, Right semicircle, from genomic characteristics to imaging phenotypes: bioinformatics analysis can reveal the pathways, biological processes, and expression of protein corresponding to specific molecular markers detected by sequencing technique. These biological processes can finally be mapped in the form of different imaging phenotypes (like necrosis or edema) on multi-modal images, and then captured by the quantitative radiomic features (such as shape, texture, or wavelet features). EGFR, Epidermal growth factor receptor; GO, Gene Ontology; GSEA, Gene set enrichment analysis; RRS, Radiomic risk score.

In addition to predicting the expression status of specific molecular markers, the current imaging genomics researches can be divided into several directions. On the one hand, researchers can first identify the prognostic imaging features (like OS or progression-free survival, PFS) to calculate individualized indicators (such as radiomic risk score, RRS), based on which patients will be further classified into different subgroups (low- or high-risk groups). Some bioinformatics analysis methods such as Gene Ontology (GO), Gene Set Enrichment Analysis (GSEA) analysis can be further used to detect differences in molecular biology of different subgroups, corresponding signal pathways, and downstream biological processes (28, 54, 55). Another research paradigm is the reverse process of the above method (13, 56). Bioinformatics analysis can reveal the pathways, biological processes, and expression of protein corresponding to specific molecular markers detected by sequencing technique. These biological processes can finally be mapped in the form of different imaging phenotypes (like necrosis or edema) on multi-modal images, and then captured by the quantitative radiomic features (such as shape, texture, or wavelet). A pioneering radiogenomics study by Zinn et al. (13) showed that radiomic-features could significantly predict periostin expression status in GB patients (Area under the receiver operating characteristic curve, AUC=76.56%) and orthotopic xenografts (AUC=92.26%). The authors tried to establish causality between radiomic texture features and expression levels in a pre-clinical model. This was one of the first radiogenomics studies highlighting a causal linkage between specific gene expression status and radiomic-features of GB. This kind of research paradigm is more suitable for the verification of the radiogenomics model and its corresponding conclusions. However, to date, it has not been elucidated how specific biological processes reflect phenotypes through imaging. Additional multidisciplinary studies in the future are needed to shed more light on this subject. **Figure 2** describes the common steps of an imaging-genomic study and **Table 2** summarizes several imaging-genomic studies in patients with GB.

**Table 2:** Applications of imaging-genomics for exploring potential signaling pathway or biological processes or in GB.

Study	Imaging Modality	General Purpose	Total Patients Training + Testing	No. of features Initial + Final		Performance AUC/Accuracy	Statistical Analysis
**Choi et al. (** 51 **)**	MRI, T1+T1CE+T2+Flair	Exploring biological characteristics behind imaging phenotypes in GB.	200 (144 + 56)	478	7	NA	Lasso-Cox, RRS, GSEA
**Itakura et al (** 57 **).**	MRI, T1CE	Verifying distinct pathway changes behind different imaging subtypes of GB.	265 (121 + 144)	388	388	NA	k-means consensus clustering, IGP
**Beig et al. (** 28 **)**	MRI, T1CE+T2+Flair	Exploring prognostic stratification-based biological processes in GB.	203 (130 + 73)	2850	25	0.84^c^	Lasso-Cox, RRS, GO, GSEA
**Park et al. (** 29 **)**	MRI,T1+T1CE+T2DWI +Flair+DSC	Identifying Core signaling pathway in GB. (RTK, p53, and RB1 pathways).	120 (85 + 35)	6472	123	(RTK) 0.88^a^	Lasso, RF, logistic regression,
**Akbari et al. (** 58 **)**	MRI, T1+T1CE+T2+Flair	Predicting status of EGFRvIII and exploring associated biological processes.	129 (75 + 54)	NA	NA	0.86^a^	Multivariate model, SVM
**Zinn et al. (** 52 **)**	MRI, T1+T1CE +Flair	TP53-PTEN-EGFR mutational landscape in GB.	29	2480	457	NA	Spearman rank correlation Hierarchical clustering
**Beig et al. (** 53 **)**	MRI, T1CE+ T2+Flair	Predicting hypoxia pathway and overall survival in GB.	115 (85 + 30)	270	8	0.83^c^	GSEA, Cox model, Unsupervised clustering
**Hsu et al. (** 59 **)**	MRI, T1CE+ DWI	Classifying immunophenotypes of GB by radiomic features.	116 (32 + 84)	9809	76	79%^b^	RF, Information gain, GSEA Logistic regression
**Beig et al. (** 54 **)**	MRI, T1CE+ T2+Flair	Identifying sex-specific biological processes and prognostic of GB.	313 (130 + 183)	2850	8	0.88^c^	Lasso-Cox, RRS, GO, GSEA
**Zinn et al. (** 13 **)**	MRI, T1CE+ T2+Flair	Validating causality between Periostin expression status and MRI-features in GB.	93 (xenografts=40)	4880	31	0.93^a^ (xenografts)	Lasso, GSEA, XGboost

DSC, Dynamic susceptibility contrast; DWI, Diffusion weighted imaging; EGFR, Epidermal growth factor receptor; Flair, Fluid-attenuated inversion recovery; GO, Gene ontology; GSEA, Gene set enrichment analysis; IGP, in-group proportion statistic; Lasso, Least absolute shrinkage and selection operator; MRI, Magnetic resonance imaging; NA, Not available; PET, Positron emission tomography; RB1, Retinoblastoma 1; RF, Random forest; RRS, Radiomic risk score; RTK, Receptor tyrosine kinase; SVM, Support vector machines; T1, T1-weighted MRI; T2, T2-weighted MRI; T1CE, contrast-enhanced T1-weighted MRI; XGboost, Extreme Gradient Boosting.

The values in the performance column were achieved using the best model in the test set. ^a^, ^b^, and ^c^ are used to mark the AUC, accuracy, and concordance index values, respectively.

Although imaging-genomics has broad application prospects, current researches mainly focus on one or several aspects of the procedures and purpose discussed above. Here, we will review the recent progress in the utilization of the imaging-genomics analysis in GB patients.

## Progress of Imaging-Genomics in GB

### Candidate Molecular Alterations

#### Isocitrate Dehydrogenase 1 and 1p/19q Codeletion

Genetic analysis revealed that the IDH1 gene mutations exist in approximately 12% of GB (21). Tumor-related IDH1 mutations result in the enhancement of enzyme function, which causes the accumulation of R(-)-2-hydroxyglutarate (2HG), and the accumulation of 2HG might drive oncogenesis (60). IDH1 mutation is also an independent factor for predicting the prognosis of patients with glioma (61), and the IDH and 1p/19q codeletion status has been introduced into the revised WHO classification of CNS tumors (6).

There have been numerous studies attempted to reveal the links between the IDH1 and 1p/19q codeletion status with the imaging-phenotypic appearance of GB (15, 38, 41–43). A model for predicting the state of molecular markers (such as IDH1) needs to take into account the distribution of data, considering that the vast majority of GB are IDH1 wild types (17). In this context, some techniques (like the synthetic minority oversampling technique (SMOTE) and adaptive synthetic) have been proven to have better performance in some cases (62). In a study of 225 GB patients by Li et al. (41), after balancing the data with the SMOTE algorithm, the model combining all‐region (necrosis, enhancement, non‐enhancement, and edema areas) radiomics features with clinical information achieved the best performance of a 97% accuracy. The radiomics model with multi-regional features has the potential to predict the IDH1 mutation status of GB patients, and the data balance is beneficial to improve the classification performance. Li and colleagues (44) used 18F-fluorodeoxyglucos PET scans from 127 patients, after feature selection through least absolute shrinkage and selection operator (LASSO), the final SVM model used 11 features and yielded an area under curve (AUC) of 90% in the validation set. In general, radiomics utilizing multimodal images seems to achieve better performance than a single modality. Nevertheless, there are also exceptions. Using a 3D-Dense-UNets approach, Yogananda et al. (63) reported a mean accuracy of 97.14% in predicting IDH status. Interestingly, the authors demonstrated similar performance when comparing the T2-based network with the multi-contrast network. One possible reason is that the deep-learning networks using conventional single-modality MR images avoid the effect of head motion due to long scan times, which helps promote clinical translation.

Choi et al. combined perfusion and conventional MRI for the prediction of IDH genotype in a group of 463 patients. The authors reported that the long short-term memory model, a type of recurrent neural network (RNN), showed performance with an accuracy of 92.8% in the validation set and 91.7% in the test set (50). Also, the RNN model can provide certain interpretability by demonstrating which temporal features are crucial for the prediction of IDH genotypes. By performing principal component analysis on their final CNN layer, Chang and co-workers (42) selected the imaging features and obtained a prediction accuracy of 94% for IDH status and 92% for 1p19q codeletion. Recently, Choi and colleagues (15) used MRI data from multiple centers to predict IDH genotypes in a cohort of 1,166 patients with glioma (WHO grade II-IV). Researchers developed an automated approach containing two CNN models, one for tumor segmentation and the other for IDH status prediction. Finally, the automated model achieved the highest diagnostic accuracy of 93.8% and 87.9% in the internal and external test set, respectively. A fully automated process is conducive to the repeatability and generalization of results, which reduces the interference of human factors and is beneficial to future application and promotion of the models.

#### O (6)-Methylguanine-DNA Methyltransferase

The MGMT gene encodes a DNA repair enzyme named O6-alkylguanine DNA alkyltransferase (64). In this process, the methylmoiety is transferred onto the MGMT protein and thus is consumed (64, 65). The epigenetic modification of the cytosine-phosphate-guanine (CpG) island at specific CpG sites of the MGMT promoter silences the gene, resulting in low DNA alkylation repair efficiency and enhanced response to temozolomide (TMZ) (66, 67).

The methylation status of the MGMT gene promoter has been considered as a crucial biomarker of tumor response to TMZ chemotherapy. Consequently, it has become an important molecular marker in imaging-genomics research (68–71). Sasaki and colleagues (72) extracted features from MRI data of 201 newly diagnosed GB patients. The diagnostic model of MGMT promoter using two significant Gray-level Co-occurrence Matrix texture features selected by LASSO only yielded a low accuracy of 67%, which remains insufficient for practical use. Wei and colleagues (45) found that the fusion radiomics signature (clinical factors and texture features) showed great performance for predicting MGMT promoter status of glioma, with an AUC value of 0.902 in the validation cohort. Moreover, the model could classify patients into low-risk and high-risk groups for overall survival (OS) after TMZ chemotherapy, which may serve as a basis for individualized treatment recommendations. Li et al. (46) predicted the MGMT promoter methylation used 1,705 radiomics features from multiregional and multiparametric MRI in multicenter cohorts. The final model achieved an accuracy of 80% in the test dataset. Distinguished from the studies in the past (43), however, this study suggested that combing clinical factors with radiomics features did not improve the prediction performance.

PET-based radiomics is also a promising method to noninvasively evaluate the MGMT status of gliomas. Qian et al. (47) extracted features from PET images of 86 patients with GB. Features were selected by comparing the weighted feature coefficient and filtering with bivariate analysis. The Random Forest model using the appropriate parameters from the grid search achieved 80% ± 10% accuracy for the prediction of MGMT promoter status. Kong et al. (73) used PET images from 107 patients with WHO grade II-IV primary diffuse gliomas for prediction. Using Wilcoxon ranksum test for features selection, a radiomics signature comprising five final features for the prediction of MGMT promoter status could be identified, yielding an AUC of 0.86 in the validation sets. However, this model lacks further validation on an external dataset.

The CNN-based classifiers achieved the accuracy from 82.7% to 94.9% in predicting the methylation status of MGMT promoter in GB (74, 75). Chen et al. (74) utilized a deep learning pipeline for automatic tumor segmentation and MGMT promoter status prediction in an end-to-end manner for GB patients. The better tumor segmentation and MGMT prediction performance both came from Fluid-attenuated inversion recovery (FLAIR) images. The CNN-based prediction model in this study had only 4 layers and achieved an accuracy of 82.7%, which might be further improved by a deeper network architecture with multi-layers. Korfiatis et al. (75) compared three different residual deep neural network (ResNet) architectures to evaluate their performance in predicting MGMT promoter status in patients with GB. The researchers found that the ResNet50 architecture (the deepest model with 50 layers) outperformed the two shallower architectures (ResNet18 with 18 layers, ResNet34 with 34 layers), and achieved an accuracy of 94.9% in the test set. Networks with deeper architectures could potentially improve the performance of the model, which also increases the risk of overfitting. Transfer learning and fine-tuning of the existing pre-trained network are alternative methods to improve the utilization of the limited data (34).

#### Other Potential Genetic/Molecular Markers

EGFR is a transmembrane tyrosine kinase that regulates normal cellular growth in epithelial cell lines. The most common EGFR mutant in GB is EGFRvIII, which is found in 31% of patients (76). Using support vector machine–based approaches, Akbari et al. (58) construct an imaging signature of EGFRvIII and achieved an accuracy of 87% in 129 patients with primary GB. The authors also reported that EGFRvIII mutant GB exhibit increased neovascularization and cell density, which is consistent with a more infiltrative phenotype. Zinn and colleagues (52) demonstrated a relationship between MRI radiomics signatures and the TP53-PTEN-EGFR mutational landscape in 29 GB patients from The Cancer Genome Atlas (TCGA). The radiomics features generated by texture analysis revealed different cellular biofunctions, such as angiogenesis, invasion, and immune response. Despite some gaps exist, glioma radiomics landscape is approaching its genomic counterpart in heterogeneity and complexity.

Diffuse midline glioma with histone H3-K27M mutation is a newly defined entity in the WHO group of grade IV diffuse gliomas (77). Those tumors carry a poor prognosis and frequently occur in brainstem, thalamus in the pediatric population. The underlying histological and radiographic variations (78) make it possible to predict the H3-K27M status of a tumor using radiomics analysis. Su and colleagues (48) performed an automated machine learning analysis on MRI data from 100 patients with midline gliomas. After feature selection and model optimization using The Tree-based Pipeline Optimization Tool (TPOT), they finally obtained an AUC of 0.903 and an average precision of 0.911 for the prediction of H3 K27M mutant status in midline glioma. Automated machine learning methods (such as TPOT) are relatively researcher-independent, which increases the robustness of the results.

### Signaling Pathways and Biological Processes

Genomic analysis has identified a highly interconnected network of aberrations in GB, including three major pathways: receptor tyrosine kinase (RTK), the retinoblastoma 1 (RB), and tumor protein p53 tumor suppressor pathways (4). Radiogenomics studies have revealed the relationship between different imaging features and certain signaling pathways or biological processes.

In a study of 120 patients with GB, using diffusion, perfusion MRI, and next-generation sequencing technology, Park and colleague (29) combined radiomic and genomic features to predict core signaling pathways in IDH wild-type GB. Different types of radiomic features were identified by the combined radiogenomics model, corresponding to RTK, P53, and RB pathways respectively. This study demonstrates that MRI features can help noninvasively identify the core signaling pathway alterations in GB, which may further support the use of targeted therapy for glioblastoma rather than a one-size-fits-all approach. Another study based on perfusion MRI showed that the elevated perfusion features were significantly associated with poor patient survival in a subgroup of GB patients. Angiogenesis and hypoxia pathways were enriched in this subgroup, suggesting the possible efficacy of anti-angiogenic therapy (79). Similarly, imaging features from enhancing tumor and edematous regions were identified to be associated with the hypoxia pathway (53), imaging markers of hypoxia can discriminate GB patients as short, mid, and long-term survivors.

Radiogenomics studies also highlight sex-related biological variations in GB patients. Colen et al. (80) revealed sex-specific molecular mechanisms for cell death (MYC oncogenic in female GB patients, TP53 apoptotic in male GB patients). Similarly, a recent study by Beig et al. (54) suggested that higher expression of texture features from enhancing tumor regions seemed to be more enriched in ‘high-risk’ group in the male population. This result appeared to be opposite in the female population, in which the same features were enriched in the ‘low-risk’ group. These findings suggesting the importance of treating sex as a covariate in the design of a radiogenomics study.

### Heterogeneity and Molecular Subtype

Intra-tumoral Heterogeneity in GB has been a topic of long-standing interest (23, 81). Spatio-temporally comprehensive assessment of the tumor heterogeneity requires multiple spatially sampling at different time points. In daily clinical practice, effective tissue sampling remains a significant challenge in accurately evaluating the intratumoral heterogeneity of a GB. Radiogenomics has shown the initial prospects for spatially evaluating the regional and genetically distinct subtypes that coexist within a single GB tumor (30, 82). By collected 48 image-guided biopsies from 13 GBs, Hu et al. (30) co-registered each biopsy location with MRI texture map to correlate spatially matched imaging measurements with regional genetic status. They identified significant imaging correlations for several driver genes, including EGFR, PTEN, RB1, TP53, and more. The author also emphasized that even within one tumor segment (such as the enhancement part in T1 contrast-enhanced MRI), regional genetic diversity can also exist. This suggests the need to improve the image-based assessment of genetic heterogeneity beyond the use of the subregion presented in a single imaging mode.

Images contain information about tumor phenotypes that are regulated not only by cell-intrinsic biological processes but also by the tumor microenvironment (59, 83–85). Recently, machine learning-based radiomics models demonstrated the capacity to evaluate enrichment levels of different immune cells in GB patients. Using radiomics features of MR images, the radiomics models could classify the immunophenotypes of GB and can predict patient prognosis (59). Lin et al. (84) used a consistent clustering method to classify radiological features into two subgroups. The two subtypes have significantly different histological stages and molecular factors (such as IDH and MGMT). Furthermore, biological information analysis hinted that the inferior prognosis subtype may be more responsive to immunotherapy. At the same time, if GB patients receive immunotherapy (such as immune checkpoint blockade therapy), the responses of therapy might also be distinguished by radiomics signatures noninvasively (86). Cho et al. (83) revealed that immune cell markers have significant correlations with perfusion and diffusion MRI features in a study of 60 GB patients. The apparent diffusion coefficient values were correlated with the expression level of immune cell markers, which can be used as an immune biomarker to predict the progression and prognosis of GB patients. It should be noted that many classification methods based on radiomics characteristics are still in an early developmental stage. Although these potential associations are encouraging, some conclusions still need further verification.

### Prognostic Stratification-Based Tumor Biological Characteristics

A large number of retrospective studies have revealed the link between the radiomics characteristics and survival time of GB patients (70, 87). According to radiomics risk stratification, some studies (28, 54, 84, 88) also tried to explore the corresponding tumor biological information. Based on survival-related imaging features, some studies (28, 54) construct individualized RRS, by which the patients will be divided into subgroups with different risk stratifications. Further, bioinformatics analysis such as GO and/or GSEA will be used to identify the signaling pathway networks or specific biological characteristics that were associated with RRS features. For example, using data from public platforms, Beig and colleagues (28) extracted 936 3D-radiomics features from T1, T2, and Flair MR images of 203 patients with GB. Finally, the GO and GSEA revealed associations of RRS signatures with signaling pathways for cell adhesion, cell differentiation, and angiogenesis. A somewhat similar approach was followed by Choi et al. (51) who used RRS weighted by Lasso-Cox model to classify patients with GB. Finally, GSEA suggested that the transcriptomic characteristics enriched in personalized subtypes were consistent with radiomics phenotypes. For example, the rim-enhancing necrotic subtype could be described by T2-derived radiomic features and further highlighted by the inflammatory genomic signatures.

Radiogenomics has the potential to unravel the molecular underpinning behind the imaging-derived phenotypes. However, these associations seem to be indirect, since they were constructed through survival variables, such as OS and PFS. Indirect associations may obscure or expand the relationships between radiomics characteristics and molecular phenotypes. In addition to using optimized AI models to integrate all such information, radiogenomics biological validation in animal models (13) would be a promising direction in the future.

### Personalized Treatment and Assessment

At present, several studies are exploring combination imaging-genomics with the assessment of early treatment response. Imaging biomarkers have shown great potential to stratify the therapeutic response of patients with recurrent glioblastoma treated with bevacizumab (89). EGFR extracellular domain mutations in GB also present opportunities for imaging and therapeutic development (56). Wei and colleagues (45) used logistic regression to generate a fusion radiomics signature based on conventional MRI of 105 patients with WHO grade II-IV astrocytomas. The fusion signature exhibited great power for predicting the methylation status of MGMT promoter and evaluating temozolomide (TMZ) chemotherapy effect. Petrova et al. (90) collected perfusion and diffusion images from 54 subjects with recurrent GB and subsequently treated with bevacizumab. Among the six different classifiers, the authors found that SVM was able to identify 97% of subjects that would respond to bevacizumab with accuracy for PFS and OS of 82% and 78%. This can help assess whether a GB patient can benefit from anti-angiogenic therapy. However, the model still needs to prove its repeatability and generalizability in an extensive cohort study.

GB is a heterogeneous disease composed of multiple molecular subtypes with different clinical courses, leading to different treatment strategies and prognoses. Radiomics and genomics attempt to explain the diversity of tumors from different perspectives. A more fused model might be used to integrate clinical information and all experimental data of an individual, and propose corresponding molecular networks specific to a personalized GB for efficient early-stage diagnosis, precise treatment recommendations, and prognostic assessment. At present, the underlying transformation of imaging-genomics remains only at an early stage of the investigation. Imaging signatures identified by animal models may be useful for future clinical trials enriched for patients with specific subtypes of GB (13, 86).

## Future Challenges

### Big Data and Data Sharing

One of the fundamental challenges in imaging-genomics research is the availability of large, standardized, well-annotated data sets. Many studies lacked external testing for evaluating the performance of the models due to the limit of data. While external testing is crucial to confirm model generalizability across different institutions. Data sharing between institutes would bring new opportunities for researchers. The Cancer Imaging Archive (TCIA, https://www.cancerimagingarchive.net/) (91), a part of the Cancer Genome Atlas (TCGA), can provide open-access cancer medical imaging and corresponding genome resources to support imaging-genomics researches (59, 63). Some information about the tumors and corresponding supporting data has been summarized on the TCIA website (https://www.cancerimagingarchive.net/collections/).

Radiological images and biological data from CPTAC (Clinical Proteomic Tumor Analysis Consortium, https://proteomics.cancer.gov/programs/cptac) (92, 93) have also been made publicly available to enable researchers to investigate the imaging phenotypes which may correlate to corresponding genomic, proteomic, and clinical data (28, 54). Additionally, the Ivy Glioblastoma Atlas Project (IvyGAP, http://glioblastoma.alleninstitute.org/) (94) provides resources for exploring the anatomic and genetic basis of GB at the radiological and molecular levels. The dataset contains RNA sequencing data, tissue sections, as well as partial MR data in TCIA. Data from these public databases has motivated and continues to motivate the technological exploration and development in imaging-genomics field. However, such data are far from sufficient. Further researches focused on developing improved algorithms (such as transfer learning or data augmentation technologies) optimized for smaller datasets are also needed.

### Quality Control, Interpretability, and Application Challenge

Even for the same data, two different calculation software might generate different feature values (95). The lack of standardized definitions will hamper the clinical use of the models. Undoubtedly, quality control and repeatability should run through the entire imaging genomic study, including data collection, preprocessing, delineation of ROIs, features extraction and selection, model building, and verification. Lambin et al. (10) have proposed the radiomics quality score (RQS) to assess the quality of radiomics studies, which plays a guiding role in improving the initial design of an imaging-genomics research. The Image Biomarker Standardization Initiative (IBSI) also produced and validated a series of consensus-based reference values for radiomics features (95). These reference values can be used to check whether a radiomics software complies with IBSI standards. Improved research quality and reproducibility can promote the clinical translation of radiomics.

In the clinical setting, there are still many obstacles to the standardized processing of data and the effective deployment of the pre-trained models. The lack of sufficient verification of a model will inevitably limit its further application. Additionally, it is also important to note that features and models generated by AI (especially deep learning) often lack clear interpretable parameters. Even if AI algorithms provide good predictive results, the models and deep features are difficult for clinicians to interpret, which limits the integration of radiomics into routine clinical practice. In the context of a regulated healthcare environment, this “black box” problem of AI algorithms is particularly outstanding. Visualization of models and features is an alternative way to alleviate this problem. Interpretability in AI is an emerging field of increased research efforts in the data science community.

### Multi-Omics Integration

Multi-omics is undergoing the big data revolution. Genomic analysis such as gene mutation or altered gene expression alone does not always reflect the real change of the corresponding protein and downstream metabolite. The intricate connections between tumor biology and holistic phenotype could have been better captured by multidimensional data and multi-omics models. In recent years, leveraging AI and digital pathology has shown significant promise in cancer diagnosis (38, 49). Remarkably, there have been attempts to integrate pathological section data and radiological data (96), which is called a new term as radopathomics. The fusion signature combining information from distinct dimensions could better predict discrepancies of treatment response. These results have exhibited the great potential of radiomics in the integration of modern precision medicine.

Predictably, the evolution of the radiomics field will gradually differentiate into “radio-…omics”, such as radio-proteomics, radiopatho-genomic, or even radio-metabonomics in the coming future, dependent on the source used. As such, current imaging-genomic association maps entail integrations of large, “multi-omic” data sets with imaging to bridge the missing links. Certainly, such a large number of omics features can easily lead to overfitting. Optimal feature reduction and selection methods and appropriate model validation are also needed to deal with the problem of “Curse of Dimensionality”.

## Conclusion

The evolution of the field of radiomics and radiogenomics is similar to the emergence of the microscope in pathology. It is foreseeable that based on multi-parameter imaging features, more biological information can be mined to evaluate the biological characteristics of GB for supporting individualized patients management. However, many challenges still exist and much work needs to be done. Better interpretability and validation of these tools will make imaging genomics more acceptable in this field. Even though the translation of these models from research to clinical practice is still in progress, they provide promise for guiding precision medicine and subsequent individualized therapeutic intervention.

## Author Contributions

HL and WZ conceived this review, organized, and critically revised the manuscript. DL and JC did major work of the manuscript. XH, KY, YL, GH, and HG made contributions to the writing and revising of the manuscript. All authors contributed to the article and approved the submitted version.

## Funding

This study was supported by grants from the National Natural Science Foundation of China (No. 81972350, 81701671), the project of Jiangsu Provincial Medical Youth Talent (No. QNRC2016047), the Medical Scientific and Technologic Development Project of Nanjing (No. ZKX15035), the Jiangsu Provincial Medical Innovation Team (No. CXTDA2017050), the Medical Science and Technology Development Foundation of Nanjing (No. ZDX16011), and the Nanjing Medical Science and technique Development Foundation (No. QRX17087).

## Conflict of Interest

The authors declare that the research was conducted in the absence of any commercial or financial relationships that could be construed as a potential conflict of interest.

## Abbreviation

AI, Artificial intelligence; AUC, Area under receiver operating characteristic curve; CNNs, Convolutional neural networks; CNS, Central nervous system; CpG, Cytosine-phosphate-guanine; EGFR, Epidermal growth factor receptor; FLAIR, Fluid-attenuated inversion recovery; GB, Glioblastomas; GO, Gene Ontology; GSEA, Gene set enrichment analysis; 2HG, R(-)-2-hydroxyglutarate; IBSI, Image biomarker standardization initiative; IDH, Isocitrate dehydrogenase; LASSO, Least absolute shrinkage and selection operator; MRI, Magnetic resonance imaging; MGMT, O(6)-methylguanine-DNA methyltransferase; NF1, Neurofibromatosis 1; OS, Overall survival; PET, Positron emission tomography; PFS, Progression-free survival; PTEN, Gene of phosphate and tension homology deleted on chromosome ten; PIK3, Phosphatidylinositol-3-OH kinase; RB1, Retinoblastoma 1; RF, Random forest; RNN, Recurrent neural network; ROI, Region of interest; RRS, Radiomic risk score; RTK, Receptor tyrosine kinase; SMOTE, Synthetic minority oversampling technique, SVM, Support vector machines; TCGA, The Cancer Genome Atlas; TCIA, The Cancer Imaging Archive; TMZ, Temozolomide; TPOT, Tree-based Pipeline Optimization Tool; WHO, World Health Organization.
